# Divergent roles for Eph and Ephrin in Avian Cranial Neural Crest

**DOI:** 10.1186/1471-213X-8-56

**Published:** 2008-05-21

**Authors:** Dan O Mellott, Robert D Burke

**Affiliations:** 1 Department of Biochemistry and Microbiology, University of Victoria, Victoria, Canada; 2 Department of Biology, University of Victoria, Victoria, Canada

## Abstract

**Background:**

As in other vertebrates, avian hindbrain neural crest migrates in streams to specific branchial arches. Signalling from Eph receptors and ephrins has been proposed to provide a molecular mechanism that guides the cells restricting them to streams. In mice and frogs, cranial neural crest express a combination of Eph receptors and ephrins that appear to exclude cells from adjacent tissues by forward and reverse signalling. The objective of this study was to provide comparative data on the distribution and function of Eph receptors and ephrins in avian embryos.

**Results:**

To distinguish neural crest from bordering ectoderm and head mesenchyme, we have co-labelled embryos for Eph or ephrin RNA and a neural crest marker protein. Throughout their migration avian cranial neural crest cells express EphA3, EphA4, EphA7, EphB1, and EphB3 and move along pathways bordered by non-neural crest cells expressing ephrin-B1. In addition, avian cranial neural crest cells express ephrin-B2 and migrate along pathways bordered by non-neural crest cells expressing EphB2. Thus, the distribution of avian Eph receptors and ephrins differs from those reported in other vertebrates. In stripe assays when explanted cranial neural crest were given the choice between FN or FN plus clustered ephrin-B1 or EphB2 fusion protein, the cells strongly localize to lanes containing only FN. This preference is mitigated in the presence of soluble ephrin-B1 or EphB2 fusion protein.

**Conclusion:**

These findings show that avian cranial neural crest use Eph and ephrin receptors as other vertebrates in guiding migration. However, the Eph receptors are expressed in different combinations by neural crest destined for each branchial arch and ephrin-B1 and ephrin-B2 appear to have opposite roles to those reported to guide cranial neural crest migration in mice. Unlike many of the signalling, specification, and effector pathways of neural crest, the roles of Eph receptors and ephrins have not been rigorously conserved. This suggests diversification of receptor and ligand expression is less constrained, possibly by promiscuous binding and use of common downstream pathways.

## Background

A defining feature of vertebrates is the neural crest (NC), a transient group of cells that originate within the dorsal neuroectoderm of the neural tube during embryonic development [1]. Following an epithelial to mesenchyme transition in which they lose affinity for the neuroectodermal epithelium, NC cells individualize and gain the capacity to migrate through the underlying mesoderm. Hindbrain cranial neural crest (CNC) cells migrate ventrally to the branchial arches, where they contribute to the development of the face, jaw, neck, and heart [2]. There are 3 discrete streams of hindbrain CNC, each separated by zones free of neural crest. The most rostral stream extends from rhombomere 2 (r2) to the first branchial arch; a second stream adjacent to r4 migrates to the second branchial arch; and the third forms a bifurcating stream that extends from r6 to the third and forth branchial arches. The patterning of the cranial neural crest is distinct from the segmentation of the trunk neural crest, where the patterning can be specifically attributed to differences between rostral and caudal halves of somites [3]

Eph family receptor tyrosine kinases (RTKs) and their membrane-anchored protein ligands, the ephrins, are thought to regulate directed migration of CNC cells. In the mouse embryo EphA4, EphB1, and EphB3 are expressed by the streams of CNC cells, whereas ephrin-B2 is distributed around the clefts dividing each branchial arch [4]. When ephrin-B2 is mutated, these streams become scattered and CNC cells invade the regions where ephrin-B2 is normally expressed. It is concluded that in CNC migration, ephrin-B2 functions primarily as a ligand to activate Eph-induced forward signaling that guides migration [4,5]. On the other hand, ephrin-B1 is expressed by the migrating neural crest cells and when it is deleted only in these cells, directional migration defects are apparent [6]. As a mutation in the PDZ binding domain of ephrin-B1 produces the same defects, it was concluded that in cranial neural crest ephrin B1 acts as a receptor that activates a PDZ mediated signaling cascade [6].

A number of Eph and ephrin genes are expressed in the hindbrain region of the early chicken embryo [7-9]. Based on these observations, we set out to determine which Eph receptors and ephrins are expressed by neural crest and which are expressed by the surrounding mesenchyme and ectoderm. We have found that avian CNC cells express Eph receptors and ephrin ligands, and an in vitro functional test indicates they can function in guiding migration. However, the roles of the ephrin-B1 and ephrin-B2 subunits are switched by comparison to those shown to function in mice. We propose a mechanism by which orthologues of Eph receptors and ephrins can become canalized to different roles during evolution.

## Results and Discussion

### Co-localization of Eph/ephrin RNA and neural crest

Although Eph receptors and ephrins are known to be expressed in the hindbrain and branchial arches, it is critical to developing a hypothesis for their functions to know which Eph receptors and ephrins are expressed by neural crest and which by surrounding head mesenchyme and ectoderm. To do this, we employed double labeling in which Eph and ephrin expression patterns were evaluated by RNA in situ hybridization and CNC cells were identified by double labeling with an antibody that recognizes a NC antigen, HNK-1. Based on findings from previous studies [7-9] as well as our own cloning experiments, we focused our analysis on ephrin-A5, ephrin-B1, ephrin-B2, EphA1, EphA3, EphA7, EphB1, EphB2, and EphB3. The stages of development examined range from 11–15, during which most of the CNC cell migration in the chicken embryo takes place [1]. Embryos probed with control, sense transcripts developed only weak background staining or no color at all (data not shown). Analysis of the CNC subpopulation associated with the first branchial arch has been excluded due to technical problems with resolving NC staining from staining in the hindbrain. We were unable to detect expression of ephrin-A5 and EphA1 (data not shown).

### Avian cranial neural crest cells express EphB3, EphA3, and EphA7

**Figure 1:**
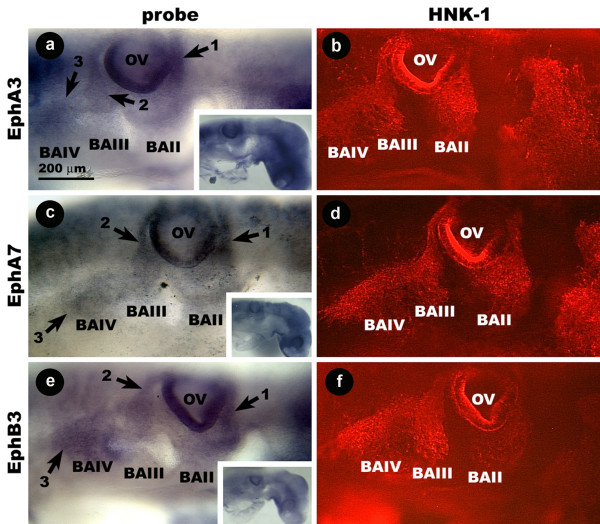
**Wholemount images showing the distribution of EphA3, EphA7, and EphB3 mRNA and HNK-1 protein in the hindbrain region of the stage 14 chicken embryo.** EphA3 probe labels streams of cells (arrows) rostral and caudal to the OV, as does the HNK-1 antibody (a, b). EphA7 (c, d) and EphB3 (e, f) probe binding cells have a similar distribution. Insets show the same embryos at lower magnification. BA, branchial arch; Ec, ectoderm; My, mesenchyme; s, somite.

EphA3, EphA7 and EphB3 mRNA localize to streams of cells on either side of the otic vesicle. In the stage 14 embryos, one of these streams reaches from BAII to the upper rostral face of the otic vesicle (Fig. 1a; arrow 1). A second reaches from BAIII to the upper caudal face of the otic vesicle (Fig. 1a; arrow 2). A third reaches from BAIV to a point in the second stream around the lower level of the otic vesicle (Fig. 1a, arrow 3). The HNK-1 antibody identifies streams of cells that have an identical distribution to those labeled by the EphA3, EphA7 and EphB3 probes (Fig. 1b–f). At stage 12 and 13, the EphB3 probe labels a stream of cells rostral to the otic vesicle. In these earlier stages, probe also labels a stream of cells caudal to the otic vesicle that appears to be the same streams before they have diverged (data not shown).

**Figure 2:**
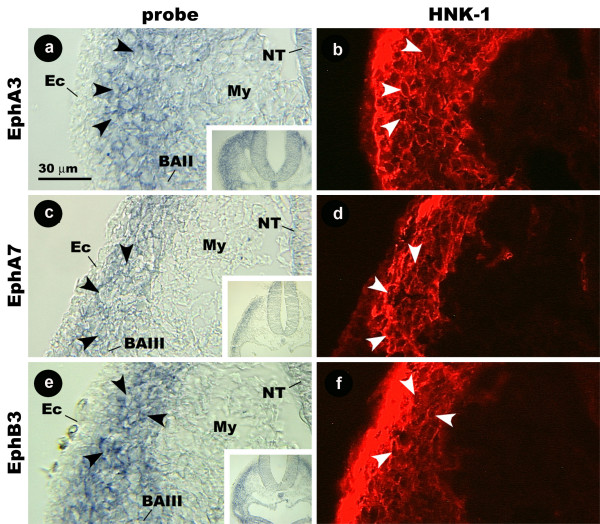
**Images of cross-sections showing the distribution of EphA3, EphA7, and EphB3 mRNA and HNK-1 protein in the hindbrain region of the stage 14 chicken embryo.** Cells labeled by EphA3 probe and HNK-1 antibody (arrowheads) form a narrow, sub-ectodermal band that reaches from the neural tube to the branchial arches (a, b). This is also the case with EphA7 (c, d) and EphB3 (e, f). Insets show the same sections at lower magnification. BA, branchial arch; Ec, ectoderm; My, mesenchyme; s, somite.

In cross-section, EphA3, EphA7 and EphB3 probe is localized to a narrow band of cells in the mesenchyme underlying the ectoderm that reaches from the neural tube to BAIII (Fig. 2a, c, d). HNK-1 staining has an identical distribution (Fig. 2b, d, f), and cells that are labeled by the probe are also labeled by the HNK-1 antibody. These results indicate that EphB3, EphA3, and EphA7 are all expressed by avian CNC cells destined for BAII, BAIII, and BAIV.

### A subset of avian cranial neural crest cells express EphA4 and EphB1

**Figure 3:**
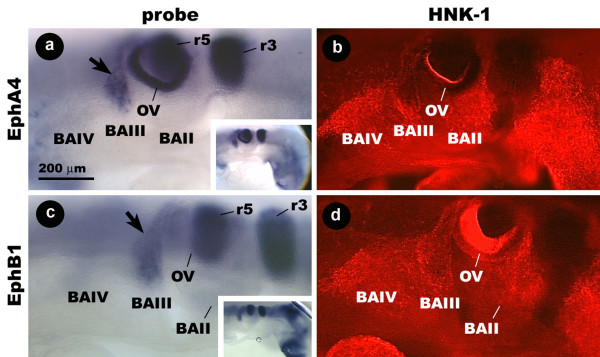
**Wholemount images showing the distribution of EphA4 and EphB1 mRNA and HNK-1 protein in the hindbrain region of the stage 14 chicken embryo.** EphA4 probe hybridizes to r3, r5, and a streak of cells caudal to the OV (arrow) that coincide with the stream of HNK-1-positive cells associated with BAIII (a, b). EphB1 probe binding cells have an identical distribution (c, d). Insets show the same embryos at lower magnification. Ec, ectoderm; My, mesenchyme; s, somite; BA, branchial arch; OV, otic vesicle.

RNA probes for Eph A4 and EphB1 hybridize most strongly to cells in r3 and r5 (Fig. 3 inset). In stage 14 embryos, probe hybridizes to a streak of cells that curls around the caudal margin of the otic vesicle and reaches into BAIII (Fig. 3a, c). Probe staining appears to coincide only with the stream of HNK-1-positive cells associated with BAIII, but not the branch associated with BAIV (Fig. 3b, d). This pattern of probe staining is also seen in stage 13 and stage 12 embryos (data not shown). At stage 11, r3/5 staining is prominent, but the post-otic vesicle stream is not obvious.

**Figure 4:**
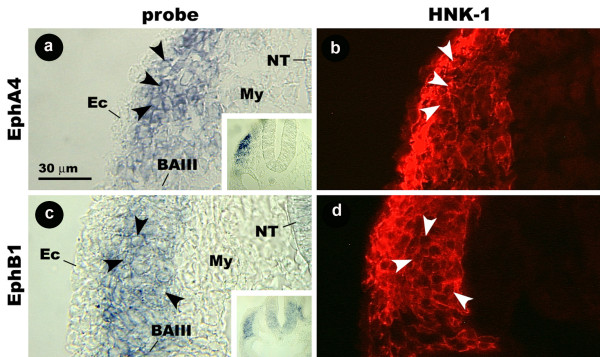
**Cross section images showing the distribution of EphA4 and EphB1 mRNA and HNK-1 protein in the hindbrain region of the stage 14 chicken embryo.** EphA4 probe binding cells overlap with cells stained by HNK-1 (arrowheads) (a, b). This is also the case with EphB1 (c, d). Insets show the same sections at lower magnification. Ec, ectoderm; My, mesenchyme; s, somite; BA, branchial arch; OV, otic vesicle.

Cross-sections reveal that EphB1 and EphA4 probes hybridize to a long, narrow band of cells in the mesenchyme immediately underlying the ectoderm between the neural tube and BAIII (Fig. 4a, c). HNK-1-positive cells have an identical distribution, and EphB1 and EphA4 probes co-localizes with the HNK-1 antibody (Fig 4b, d). From these results, we have concluded that CNC in the avian embryo expresses EphB1 and EphA4, but this expression is restricted to those cells that migrate to BAIII.

### Ephrin-B1 is expressed by cells bordering the streams of avian cranial neural crest

**Figure 5:**
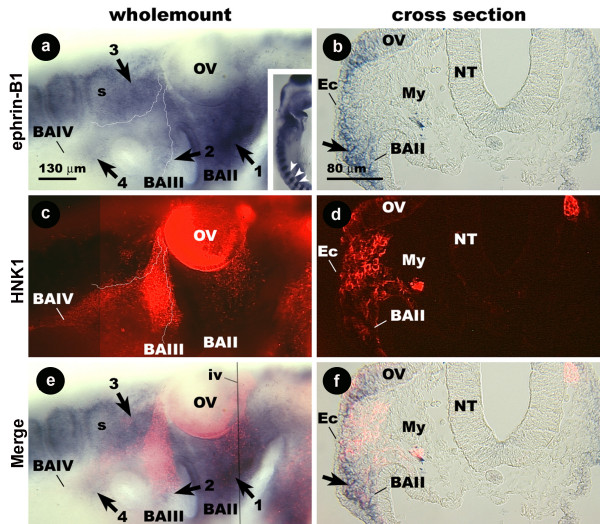
**The distribution of ephrin-B1 mRNA and HNK-1 protein in the embryonic avian hindbrain.** Wholemount (a, c, e) and cross-section (b, d, f) images of a stage 14 chicken embryo double labeled for ephrin-B1 and HNK-1. Inset shows low magnification image of staining in somites of this embryo. (a-c) The stream of CNC cells rostral to the OV comes to an end at the point where strong ephrin-B1 probe staining appears in BAII (arrow 1). The margins of the regions expressing ephrin-B1 have been traced with a thin white line in panel a. The tracing from panel a has been superimposed on the image in panel c to show the margins relative to the HNK-1 expressing cells. (c – f) The CNC stream caudal to the OV is bordered by groups of cells (arrows 2–4) labeled by the ephrin-B1 probe. In cross-section (taken at fine black line in e), probe (arrow) and HNK-1 stained cells have a non-overlapping distribution. BA, branchial arch; Ec, ectoderm; My, mesenchyme; NT, neural tube; s, somite; OV, otic vesicle.

Ephrin-B1 probe hybridizes most prominently to the caudal halves of somites (Fig. 5a inset). In stage 15 embryos, probe staining is also found in cells within BAII, and staining is strongest in the arch (Fig 5a; arrow 1). This region roughly corresponds to the same point where the stream of HNK-1-positive cells rostral to the otic vesicle comes to an end (Fig. 5b). Ephrin-B1 probe also hybridizes weakly to a thin, diffuse strip of cells that extends from the caudal surface of the otic vesicle to the cleft between BAII and BAIII (Fig. 5a, arrow 2). A second region of diffuse staining is caudal to the otic placode and extends to the first somite (Fig 5a, arrow 3). In combination, the boundaries of these 2 regions resemble an inverted v (Fig. 5a, fine outline). Probe also hybridizes weakly to a small circle of cells around the opening to BAIV (Fig. 5a arrow 4). In co-localizations it is apparent that the stream of HNK-1-positive cells caudal to the otic vesicle is bracketed by ephrin-B1 probe binding cells (Fig. 5a–c, fine outline). Similar distributions are seen in embryos at stages 12–14, although some probe binding cells are not present before stage 14 (data not shown).

In cross-section, the ephrin-B1 probe binding cells are concentrated within the ventral mesenchyme of BAII (Fig. 5d). The HNK-1 antibody binds to a band of cells distributed between the lower margin of the otic vesicle and the opening of BAII (Fig 5e). These cells become diffuse ventrally as ephrin-B1 probe staining becomes prominent within BAII. We have therefore concluded that ephrin-B1 has a distribution that for the most part, does not overlap with CNC in the avian embryo and is expressed by cells that border the pathways of CNC migration.

### Avian cranial neural crest cells express ephrin-B2

**Figure 6:**
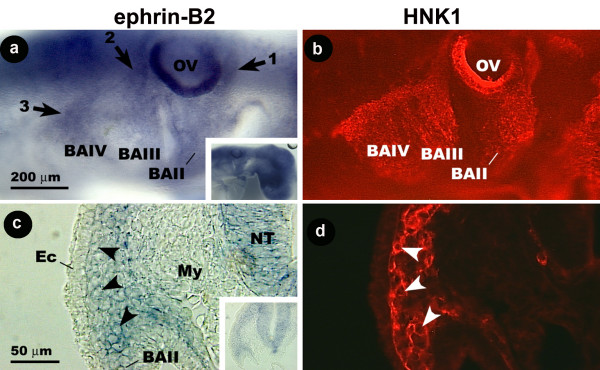
Distribution of ephrin-B2 mRNA and HNK-1 protein in the hindbrain region of the stage 12–14 embryos. At stage 14, ephrin-B2 probe labels streams of cells rostral and caudal to the otic vesicle (arrows), as does HNK-1 (a, b). The probe has a similar distribution in stage 13 (c) and stage 12 (d) embryos. Insets show the same embryos at lower magnification. In cross section, ephrin-B2 probe binding cells again co-localize with cells stained by HNK-1 (arrowheads) (e, f). Inset shows the same section at lower magnification. BA, branchial arch; Ec, ectoderm; My, mesenchyme; NT, neural tube; OV, otic vesicle. Distribution of ephrin-B2 mRNA and HNK-1 protein in the embryonic avian hindbrain. Wholemount (a, b) and cross-section (c, d) images of a stage 14 chicken embryo double labeled for ephrin-B2 and HNK-1. (a, b) Ephrin-B2 probe labels cells rostral and caudal to the OV (arrows), as does HNK-1. Inset shows embryo at lower magnification. (c, d) In cross-section, ephrin-B2 probe binding cells again colocalize with cells stained by HNK-1 (arrowheads). Inset shows section at lower magnification. Ec, ectoderm; My, mesenchyme; s, somite.

Cells labeled by ephrin-B2 probe have a distribution similar to that of cells labeled by EphB3, EphA3, or EphA7 probe (Fig. 6a). In stage 14 embryos, ephrin-B2 probe stains a stream of cells that spans from the upper rostral face of the otic vesicle to BAII. A second reaches from a point around the upper caudal margin of the otic vesicle to BAIII. A shorter region of probe binding cells branches off of this second stream around the lower level of the otic vesicle and extends to BAIV. HNK-1 has an identical distribution in the same region (Fig. 6b). This pattern of probe staining is also seen in the stage 13 embryos (data not shown). At stage 12, probe stains a short stream of cells rostral to the otic vesicle and a second caudal to the otic vesicle (data not shown).

In cross-section, the ephrin-B2 probe and HNK-1 antibody label a thin band of cells beneath the ectoderm that reaches from the neural tube to BAII (Fig. 6c, d). Therefore, ephrin-B2 is expressed by the CNC cells in the avian embryo that are destined for BAII, BAIII, and BAIV, as was the case with EphB3, EphA3, and EphA7.

### EphB2 is expressed by cells bordering the streams of avian cranial neural crest

**Figure 7:**
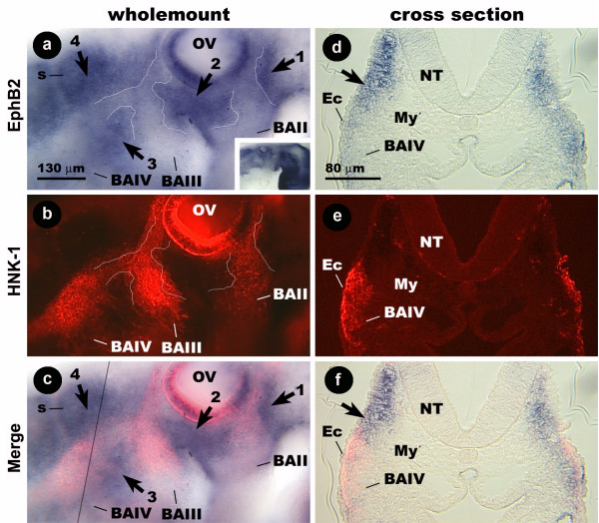
**Distribution of EphB2 mRNA and HNK-1 protein in the embryonic avian hindbrain.** Wholemount (a-c) and cross-section (d-f) images of a stage 14 chicken embryo double labeled for EphB2 and HNK-1. (a-c) EphB2 probe staining is in patches (arrows) bordering cells labeled by HNK-1. Inset shows embryo at lower magnification. The margins of the regions expressing EphB2 have been traced with a thin white line in panel a. The tracing from panel a has been superimposed on the image in panel b to show the margins relative to the HNK-1 expressing cells. (d-fi) In cross-section (taken at black line in c), probe (arrow) and HNK-1 stained cells have a non-overlapping distribution. Inset shows section at lower magnification. Ec, ectoderm; My, mesenchyme; s, somite.

In the stage 15 embryos, EphB2 probe hybridizes weakly to a narrow band of cells that extends dorsally from the cleft between BAI and BAII, leaving a thin space of unstained tissue next to the otic vesicle (Fig 7a; arrow 1). Ventral to the otic vesicle, a broader band of probe staining is positioned over the cleft between BAII and BAIII (Fig 7a, arrow 2). A third, roughly triangular patch of labeled cells is found over the cleft between BAIII and BAIV (Fig 7a, arrow 3). A region of probe staining caudal to the otic vesicle extends to the first somite (Fig 7a, arrow 4). The BAII HNK-1 positive cells largely fill the region between the otic vesicle and the boundaries of the EphB2 expressing cells (Fig 7a, b). The HNK-1 positive cells caudal to the otic vesicle occupy the spaces between the otic vesicle and the regions expressing EphB2 that approximate the shape of an inverted y (Fig. 7a, b). Similar distributions are seen in stage 13 and 14 embryos (data not shown).

In cross-section, the patch of EphB2 probe staining is in the mesenchyme underlying the ectoderm proximal to the neural tube (Fig. 7d). HNK-1 staining in the same section appears in the mesenchyme in and around BAIV, distal to the neural tube (Fig. 7e, f). Thus, HNK-1 is least concentrated where the EphB2 probe staining is most prominent. Therefore, CNC cells in the avian embryo have distributions that for the most part do not overlap with EphB2, and they appear to migrate along pathways bordered by cells expressing this receptor.

However, there are differences in the expression patterns for ephrin-B1 and EphB2 in non-neural crest cells. For instance, ephrin-B1 expression is concentrated inside of BAII (Fig. 5a, c), whereas EphB2 has a more dorsal distribution in the same region (Fig. 7a, c). Similarly, ephrin-B1 expression caudal to the otic vesicle is focused around the dorsal half of the CNC stream in that region (Fig. 5a, c), whereas EphB2 expression brackets the CNC more ventrally around BAIII and BAIV (Fig. 7a, c).

These data indicate that a suite of Eph receptors and ephrin ligands are expressed in the hindbrain region of the avian embryo during the course of CNC cell migration. RNA probes for EphA3, EphA4, EphA7, EphB1, and EphB3 label segregated streams of HNK-1 expressing cells that span the distance from the hindbrain to the branchial arches. We have therefore concluded that avian CNC cells express EphA3, EphA4, EphB1, and EphB3 and ephrin-B2. These CNC streams are positioned between groups of HNK-1-negative cells labeled by probes for ephrin-B1 or EphB2. Thus, avian CNC cells migrate along pathways bordered by cells expressing potential binding partners for EphA3, EphA4, EphA7, EphB1, and EphB3 or ephrin-B2. This led us to hypothesize that CNC cells in the avian embryo are channeled into streams and guided to their destinations by being excluded from ephrin-B1 or EphB2-expressing territories.

### Proteins that bind to ephrin-B1 and EphB2 are expressed by avian cranial neural crest cells in vitro

**Figure 8:**
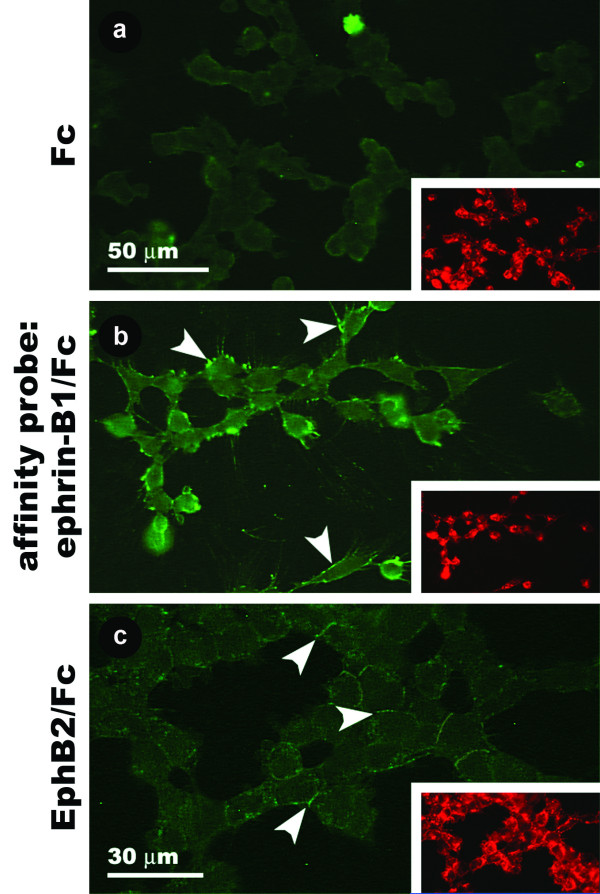
**Binding of ephrin-B1 and EphB2 affinity probes to cultured avian CNC cells.** (a) Cells exposed to Fc protein are not labeled by an anti-Fc antibody. (b) Bright peripheral spots of anti-Fc staining result from exposure of cells to ephrin-B1/Fc protein (arrowheads). (c) Cells exposed to EphB2/Fc protein exhibit weaker anti-Fc staining at points of contact between neighboring cells (arrowheads). Insets show HNK-1 staining for each field of cells.

In order to confirm that explanted avian CNC cells express proteins that interact with ephrin-B1 and EphB2, we exposed the outgrowth from cultured stage 10–12 hindbrain explants to affinity probes comprised of the extracellular domain of either ephrin-B1 or EphB2 fused to the Fc domain of human IgG and then stained them with an FITC-conjugated antibody specific for the Fc domain of human IgG. In the control experiment, where cells were exposed to the Fc domain of human IgG alone, cells co-labeled with the HNK-1 antibody fail to bind the anti-Fc antibody (Fig. 8a). Conversely, exposure to ephrin-B1/Fc fusion protein results in HNK-1-positive cells showing small, intense foci of anti-Fc staining around the cell periphery (Fig. 8b). HNK-1 positive cells exposed to EphB2/Fc also stain, but in this case it is fainter than with ephrin-B1/Fc and most prominent at the interfaces between adjacent cells (Fig. 8c). Thus, explanted avian CNC cells bind ephrin-B1 and EphB2, consistent with our localization data.

### Avian cranial cells respond to ephrin-B1 and EphB2 in a stripe assay

**Figure 9:**
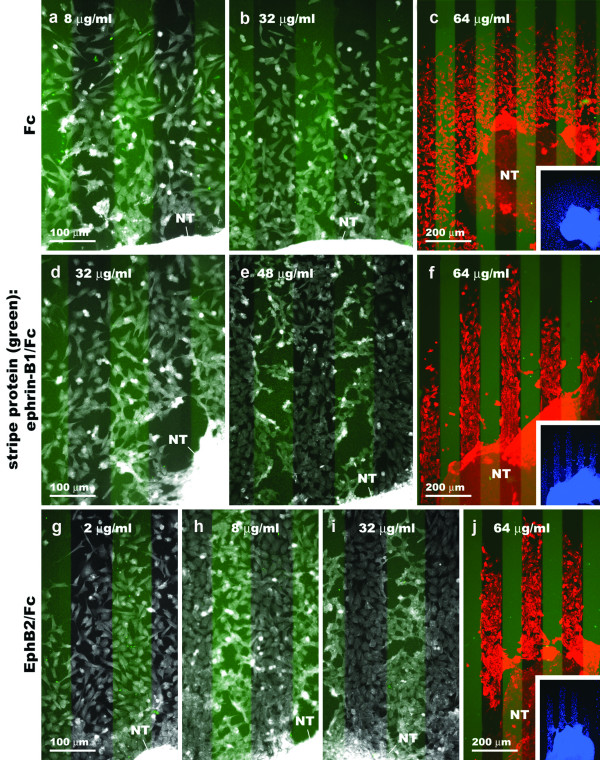
**Outgrowth of cranial neural crest cells from stage 10–12 chicken neural tube explants onto ephrin-B1 or EphB2 stripe assay substrates.** Representative results from the three experimental conditions. The position of the first set of lanes is revealed by an FITC marker (faint green stripes). In the last image on each row (c, f, j), cells have been double labeled with HNK-1 antibody (red) and DAPI nuclear stain (blue). (a-c) Clustered Fc + FN vs. FN. Cell outgrowth shows no bias for either set of lanes, regardless of Fc protein concentration. (d – f) Clustered ephrin-B1/Fc + FN vs. FN and (g – j) clustered EphB2/Fc + FN vs. FN. Cell outgrowth is even at low stripe protein concentrations, but becomes more restricted to the lanes between the FITC marked stripes with increasing concentrations in the coating solution.

To test the functions of ephrin-B1 and EphB2 in avian CNC migration, we performed a series of stripe assay experiments with these proteins. In the control experiment, stage 10–12 hindbrain explants were cultured on a substrate made up of lanes of FN alternating with lanes of FN plus an FITC marker and Fc protein pre-clustered with an anti-Fc antibody. Using Fc protein concentrations in the coating solution ranging from 8 to 64 μg/ml, HNK-1-positive cell outgrowth is always spread evenly across the two sets of lanes (Fig. 9). In contrast, substrate protein concentration has an effect on outgrowth pattern when ephrin-B1/Fc is used in place of Fc in the stripe assay. At 32 μg/ml in the coating solution, the outgrowth is even (Fig. 9). When coated with 48 μg/ml, outgrowth shows a bias for the lanes between the FITC-marked stripes, but there are also a significant number of cells found on these stripes (Fig. 9). At 64 μg/ml in the coating solution, this bias is much stronger and there are almost no cells found on the FITC stripes (Fig. 9). In the corresponding experiments with EphB2/Fc, cell outgrowth also becomes increasingly restricted to the lanes between the FITC-marked stripes with increasing substrate protein concentrations (2 to 64 μg/ml) (Fig. 9). The total cell outgrowth in these experiments is comprised almost entirely of cells labeled by the HNK-1 antibody (as revealed by DAPI nuclear staining) (Fig. 9).

**Figure 10:**
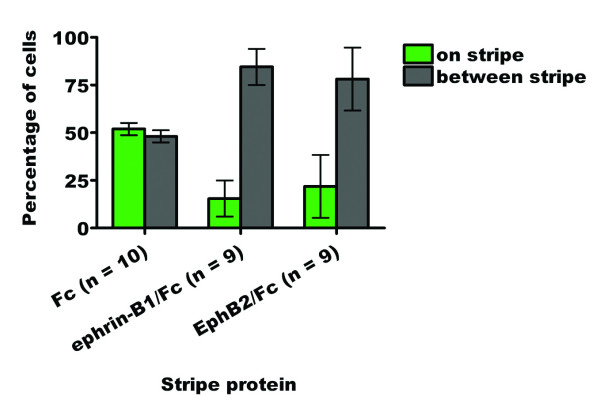
Bar graph summarizing the quantified stripe assay results for the outgrowth of cranial neural crest cells from stage 10–12 chicken neural tube explants onto ephrin-B1 or EphB2 stripe assay substrates (64 μg/ml coating concentration).

Outgrowth on the control substrate (64 μg/ml) has an essentially even distribution, with 52% of cells being found on the FITC stripes and 48% between them (n = 10) (Fig. 10). Conversely, outgrowth is highly skewed on the substrate containing lanes of ephrin-B1/Fc, with 15% of cells being found on the FITC stripes and 85% between them (n = 9). Outgrowth on the substrate containing lanes of EphB2/Fc has a similar distribution, with 22% of cells being found on the FITC stripes and 78% between them (n = 9). The difference between the control and ephrin-B1/Fc or EphB2/Fc results is significant (p < 0.0001), whereas the difference between the ephrin-B1/Fc and EphB2/Fc results is not significant (p = 0.2745). We have therefore concluded that HNK-1-positive cells growing out from avian neural tube explants in culture are indifferent to Fc alone in the stripe assay, but avoid ephrin-B1/Fc and EphB2/Fc strongly and to a similar extent.

### Soluble ephrin-B1 and EphB2 mitigate the avoidance response

**Figure 11:**
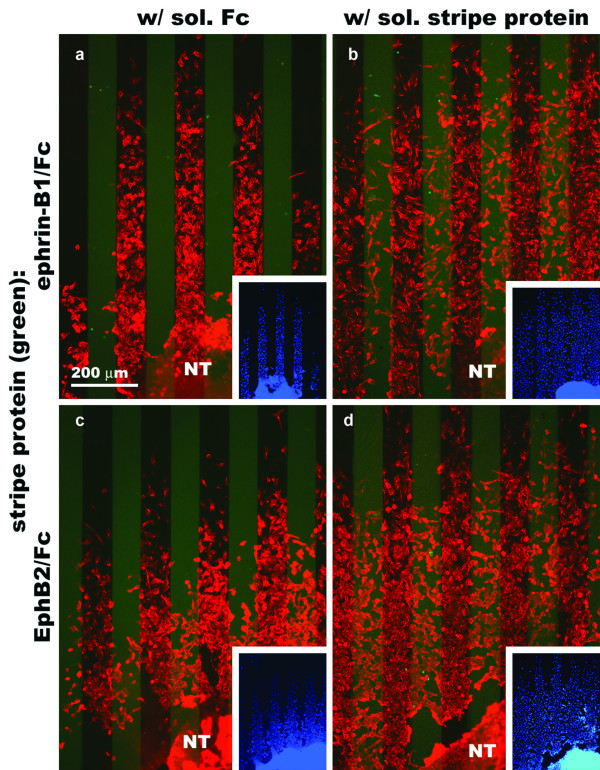
**Outgrowth of cranial neural crest cells from stage 10–12 chicken neural tube explants onto ephrin-B1 or EphB2 stripe assay substrates in the presence of soluble competitors.** Representative results from the four experimental conditions. The position of the first set of lanes is revealed by an FITC marker (faint green stripes). Cells have been double labeled with HNK-1 antibody (red) and DAPI nuclear stain (blue). All experiments were done with a stripe protein concentration of 64 μg/ml. (a). Clustered ephrin-B1/Fc + FN vs. FN with soluble Fc. Cell outgrowth from the neural tube is strongly localized to the lanes between stripes of FITC-marked ephrin-B1/Fc. (b) Clustered ephrin-B1/Fc + FN vs. FN with soluble ephrin-B1/Fc. Substitution of the soluble Fc with ephrin-B1/Fc results in an increase in the number of cells found on the stripes of immobilized ephrin-B1/Fc. (c) Clustered EphB2/Fc + FN vs. FN with soluble Fc and (d) clustered EphB2/Fc + FN vs. FN with soluble EphB2/Fc. As with the ephrin-B1/Fc experiments, there is a significant increase in the number of cells on lanes of substrate-bound EphB2/Fc in going from adding soluble Fc to soluble EphB2/Fc to the culture medium.

To determine whether the responses observed in the stripe assays are mediated by the Eph receptors and ephrin ligands expressed by CNC cells, we did the same experiments, but added soluble fusion proteins as competitors (Fig. 11). In the first control experiment, stage 10–12 neural tube explants were cultured in the presence of soluble Fc protein on a substrate composed of lanes of clustered ephrin-B1/Fc plus FN and an FITC marker alternating with lanes of FN alone. The resulting HNK-1-positive outgrowth is localized to the lanes between the FITC marked stripes of ephrin-B1/Fc protein, such that there are almost no cells on these stripes (Fig. 11). When the same experiment is done with soluble ephrin-B1/Fc added to the culture medium, there is a marked increase in the number of cells on the lanes of substrate-bound ephrin-B1/Fc. In the corresponding experiments with EphB2/Fc, there is a similar increase in the number of cells on the lanes of substrate-bound EphB2/Fc. With both sets of experiments, the results from the control are similar to the assays in which no soluble protein was added to the culture medium. It should also be noted that soluble ephrin-B1/Fc or EphB2/Fc did not completely eliminate the striped outgrowth seen in the presence of soluble Fc alone, but made it less conspicuous. As in the experiments where no soluble proteins were added to the culture medium, the total cell outgrowth from the explanted avian neural tubes is dominated by HNK-1-positive cells.

**Figure 12:**
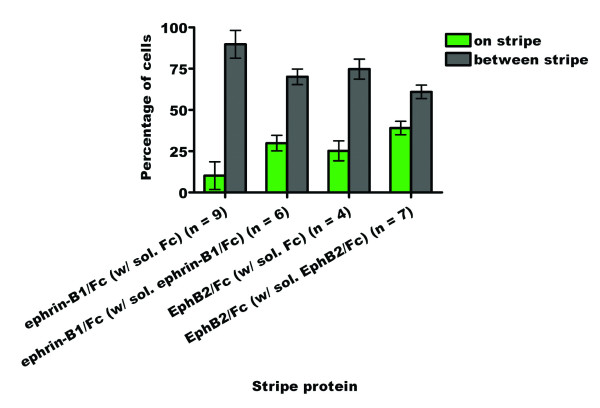
**Bar graph summarizing the stripe assay results for the outgrowth of cranial neural crest cells from stage 10–12 chicken neural tube explants onto ephrin-B1 or EphB2 in the presence of soluble competitors.** There are significantly more cells found growing on lanes of substrate bound ephrin-B1/Fc or EphB2/Fc protein in the presence of soluble competitor than in the presence of soluble Fc.

When neural tube explants are cultured on the clustered ephrin-B1/Fc plus FN and FITC versus FN substrate in the presence of soluble Fc protein, 10% of the outgrowing cells are found on the FITC stripes while 90% are found between them (n = 9) (Fig. 12). In the corresponding experiment with soluble ephrin-B1/Fc, this distribution shifts to 31% of the cells being found on the stripes and 69% between them (n = 6). When explants are cultured on the clustered EphB2/Fc plus FN and FITC versus FN substrate in the presence of soluble Fc, 25% of the outgrowing cells are found on the FITC stripes while 75% are found between them (n = 4). In the presence of soluble EphB2/Fc, this distribution shifts to 39% of the cells being found on the stripes and 61% between them (n = 7). The difference between the absence and presence of soluble Fc in the culture medium is not significant for either the ephrin-B1/Fc (p = 0.03928) or EphB2/Fc (p = 0.7390) experiments. For the soluble competitor assays, however, the difference between the control and experimental treatments is significant for both the ephrin-B1/Fc (p = 0.0004) and EphB2/Fc (p = 0.0483) assays. These results indicate addition of soluble forms of ephrin-B1/Fc and EphB2/Fc interferes with CNC exclusion from substrates coated with clustered ephrin-B1 EphB2.

These results indicate that ephrin-B1 and EphB2 can alter the direction of migration of avian CNC cells. Addition of soluble competitor resulted in a significantly more even pattern of outgrowth than in controls, presumably because the Ephs and ephrins expressed by the outgrowing cells were rendered less sensitive to the substrate-bound ephrin-B1 or EphB2 through their interactions with the soluble proteins. Taken together, these results indicate that avian CNC cells express proteins that bind to ephrin-B1 and EphB2 and mediate the repulsion of these cells from ephrin-B1 and EphB2 in the stripe assay.

This repulsion could explain how avian CNC cells are guided to their targets in vivo, given that the stereotypical pathways through which they migrate are bordered by cells expressing ephrin-B1 or EphB2. Furthermore, while the paradigm for Eph/ephrin function in directed cell migration is that Eph-expressing migratory cells or outgrowing axons are guided to their targets by receptor-mediated repulsion from ephrin-expressing cells, our results suggest that reverse signaling from Eph to ephrin can also play a role. Thus, Ephs and ephrins can regulate the path finding behavior of migrating avian CNC cells.

A remarkable aspect of these studies is the differences that are apparent within the vertebrates in the distribution of hindbrain Ephs and ephrins (Table 1). Although data are incomplete, it is apparent that the combination of Eph receptors and ephrins expressed by the different populations of hindbrain neural crest varies between the classes of vertebrates. Overall, hindbrain neural crest of mice, chickens and frogs express similar subsets of Eph receptors and ephrins, however, few of the receptors or ephrins are expressed by precisely the same populations of neural crest or bordering tissue. The patterns of hindbrain neural crest migration are similar, yet the molecules that are in part responsible for patterning the streams appear to differ. This is particularly striking with the data from mouse and chick with respect to ephrin-B1 and ephrin-B2. In mice ephrin-B1 is expressed by migrating CNC cells and there is direct evidence that it functions as a reverse signaling receptor to regulate CNC path finding [6]. In chicks, ephrin-B1 is expressed by mesoderm cells that border the migratory pathways and it appears to function as a ligand that mediates repulsion of migrating neural crest. Similarly, ephrin-B2 is expressed by all three streams of neural crest cells in chicks, yet in mice ephrin-B2 functions as a ligand and is expressed in tissues bordering neural crest pathways [4]. Ephrin-B1 and ephrin-B2 appear to have evolved opposite roles in guidance of chicken and mouse embryos hindbrain neural crest.

**Table 1:** Comparison of expression of Eph receptor and ephrin in branchial arches (BA) of 3 vertebrates

**Gene**	**Organism**	**BA I**	**BA II**	**BA III**	**BA IV**	**Reference**
***Eph B1***	*Frog*			NC/Meso	NC/Meso	12
	*Chick*			NC		This study
	*Mouse*	NC	NC	NC		3
***Eph B2***	*Frog*					
	*Chick*		Meso	Meso	Meso	This study
	*Mouse*					
***Eph B3***	*Frog*					
	*Chick*		NC	NC	NC	This study
	*Mouse*	NC	NC	NC		3
***Eph A4***	*Frog*			NC/Meso		12
	*Chick*			NC		This study
	*Mouse*		NC	NC		25
***ephrin-B1***	*Frog*					
	*Chick*		Meso	Meso	Meso	This study
	*Mouse*			NC	NC	5
***ephrin-B2***	*Frog*		NC/Meso			12
	*Chick*		NC	NC	NC	This study
	*Mouse*		Ecto/Meso	Ecto/Meso		3

Neural crest is a hallmark of vertebrates and many aspects of neural crest development are shared features. There are numerous examples of conservation of signaling pathways, transcription factors specification, and effectors genes in vertebrate neural crest [10,11]. Reviews of neural crest frequently do not distinguish data from fish, frogs, birds, and mammals, as conservation of pathways and regulatory networks in neural crest is commonplace [12]. Why do the critical roles of Eph receptors and ephrins in guiding migration appear to be unconstrained by phylogeny? It may be that although there are multiple orthologous Eph receptors and ephrins, they bind promiscuously and they activate similar effector pathways. Diversification of expression patterns then results from a process of canalization in which similar receptor-ligand binding partners activate similar downstream events making migration independent of the precise receptor or ligand expressed. For example, if ancestrally ephrin-B1 and ephrin-B2 were both expressed by neural crest and head mesenchyme and the downstream signal transduction mechanisms were the same for both subunits, in one lineage expression of ephrin-B2 becomes fixed in neural crest, whereas in another lineage expression of ephrin-B2 becomes fixed in bordering mesenchyme. It may be significant that in *Xenopus *ephrin-B2 is expressed by both BAII neural crest cells and the mesoderm along the BAII pathway [13]. The hypothesis that the Eph receptors and ephrins are interchangeable is supported by data on binding affinities of the subunits [14]. However it is not known if similar signal transduction pathways are activated by orthologous Eph receptors and ephrins in different populations of neural crest. Comparative data on the molecular mechanisms of hindbrain neural crest patterning has the potential to contribute to a more complete picture of vertebrate evolution.

## Conclusion

We have shown that Eph receptors and ephrin ligands contribute to avian hindbrain neural crest cell pathfinding, as is the case in mouse and *Xenopus*. Avian hindbrain neural crest cells express a set of Eph receptors and migrate along pathways bordered by cells expressing ephrin-B1. In addition, avian CNC cells also express ephrin-B2 and migrate along pathways bordered by cells expressing a binding partner, EphB2. Our functional analyses show that EphB2 and ephrin-B1 have repulsive effects on avian CNC cells and suggest that these effects are mediated by Ephs and ephrins. The pattern of expression of Eph receptors and ephrins by different populations of hindbrain neural crest in vertebrates appears to be less constrained phylogenetically than other neural crest signalling and specification molecules. This may be a consequence of diversification of receptor and ligand expression that was unimpeded because of promiscuous binding affinities and common downstream events.

## Methods

### 4.1 Probe plasmids and RNA in situ hybridization

To prepare probes for EphB1 and EphB2, small blocks of tissue bracketing the otic vesicle region were excised from stage 13–15 embryos. Poly(A)+ mRNA was extracted from this tissue with a MicroPoly(A) Pure kit (Ambion) and first strand cDNA was generated from the mRNA with a ProSTAR RT-PCR kit (Stratagene). Degenerate codehop primers [15] designed against conserved protein sequences of B-type Eph receptors, EphB-F1 (5'-CATCCTGGTGAACTCCAACCTNGTNTGYAARG-3') and EphB-R1 (5'-TGGATGTGGTTCAGGATCTTCTTYTGRTGNCC-3'), were then used to amplify fragments of EphB receptors from the first strand cDNA by PCR. These fragments were cloned with a TOPO-TA Cloning kit (Invitrogen). Two of the resulting clones were identified as fragments of the chicken EphB1 and EphB2 genes. In a second round of PCR, primers EphB1-F1 (5'-GAATTCGGATCTTCTTCTGGTGCCCA-3') and EphB1-R1 (5'-GAATTCGGATCTTCTTCTGGTGCCCA-3') were used to add EcoRI and PstI cut sites to the ends of the EphB1 fragment. This reaction product was subsequently cloned between the EcoRI and PstI sites of pBluescript II SK +. Likewise, primers EphB2-F1 (5'-CTCGAGTGGACAGCGCCTGAGGCAAT-3') and EphB2-R1 (5'-ACTAGTGTTCAGAATTTTCTTCTGGT-3') were used to add SpeI and XhoI sites to the EphB2 fragment so that it could be cloned between the same sites in pBluescript II KS +.

To prepare probes for EphA3, EphA7, and EphB3, neural tubes from stage 10–12 chicken embryos were isolated and grown overnight in culture (see below). cDNA was extracted from the neural tubes and their associated cell outgrowths with a Cells-to-cDNA II kit (Ambion) and used as template in PCRs with gene-specific primers. The products from these reactions were cloned with a TOPO-TA Cloning kit. Primers EphA3-F1 (5'-GTCGACCTGGGCACTTGCAAAGAG-3') and EphA3-R1 (5'-GGTACCGCCAGCATTACACAAGCAC-3') were used to amplify a fragment of the chicken EphA3 gene that was subsequently subcloned between the BamHI and EcoRV sites of pBluescript II SK +. Primers EphA7-F1 (5'-GAGCTCTACTACAAGAAGTGCTGGTC-3') and EphA7-R1 (5'-GGTACCGAGGACTCCACTCTAAACTC-3') were used to amplify a fragment of the chicken EphA7 gene that was subsequently subcloned between the KpnI and SacI sites of pBluescript II SK +. Finally, primers EphB3-F1 (5'-GAGCTCCCTGCAAAGAGACCTTCAAC-3') and EphB3-R1 (5'-GGTACCTTCATGGCTGGCTCGTAC-3') were used to amplify a fragment of the chicken EphB3 gene that was subsequently subcloned between the EcoRV and PstI sites of pBluescript II SK +.

Plasmids containing chicken ephrin-B1 [16] and EphA4 [17] sequences (provided by E. Pasquale) were used as templates in PCRs to isolate shorter fragments of the same sequences. The products from these reactions were cloned with a TOPO-TA Cloning kit. The ephrin-B1 fragment amplified by primers ephrin-B1-F1 (5'-ATCGATGAGTGGGAAAGGGTTGGTC-3') and ephrin-B1-R1 (5-GTCGACGTTGTCTGCCTCCTTGCTG-3') was subcloned between the ClaI and SalI sites of pBluescript II SK +. The EphA4 fragment amplified by primers EphA4-F1 (5'-GAGCTCTTCGTGGCATCGGCTCAGGA-3') and EphA4-R1 (5'-GAATTCCTGGAGCTCTCGCTGCCTGT-3') was subcloned between the EcoRI and SacI sites of pBluescript II SK +.

Plasmid containing chicken ephrin-B2 sequence was provided by K. Patel [18]. A PstI/SacI restriction digest fragment of this sequence was cloned between the same cut sites in pBluescript II SK +.

Probe plasmids for chicken ephrin-A5 and EphA1 (also known as EphA9) were provided by P. Antin [9].

RNA in situ hybridizations with chicken embryos ranging from stage 11 to 15 were carried out essentially as described by Streit and Stern [19].

### 4.2 Antibody labeling and sectioning after in situ hybridization

Embryos stained by the in situ hybridization procedure were incubated overnight at 4°C with HNK-1 primary antibody (ATCC) diluted to 1:100 in a blocking buffer containing 5% lamb serum. After being thoroughly washed, the embryos were incubated overnight at 4°C with Alexa Fluor 568 goat anti-mouse secondary antibody (Molecular Probes) diluted to 1:400 in blocking buffer. Embryos exhibiting strong HNK-1 and probe labeling after a final series of washing steps were prepared for sectioning by being embedded in OCT compound (Sakura) and flash frozen in liquid nitrogen. 10 μm cross-sections were collected with a cryostat, placed onto gelatin-coated slides, and rehydrated with a mounting medium comprised of 1:1 PBS and glycerol.

### 4.3 Cell culture

Neural tube explants were prepared following Newgreen and Murphy [20]. Stage 10–12 chicken embryos were harvested in PBS and dissected to remove the extraembryonic membranes, head, and tail, leaving a small block of tissue around the otic vesicle. Tissue pieces were rinsed briefly, transferred to a solution of 400 μg/ml Dispase I neutral protease (Roche) in PBS, incubated at 37°C for 30 minutes, and then transferred to a 35 mm Petri dish flooded with PBS containing 5% fetal bovine serum (FBS) (Gibco). Using fine tungsten needles, the neural tubes were gently teased away from the surrounding tissue, including endoderm, ectoderm, somites, and head mesenchyme (whenever possible, the notochord was left intact to mark the ventral surface of the neural tube). The isolated neural tubes were rinsed briefly in a cell culture medium consisting of D-MEM/F-12 with 100 units/ml penicillin, 100 μg/ml streptomycin, 1 mM MEM sodium pyruvate, 2 mM L-glutamine, 5% FBS (Gibco), and 1% OPI media supplement (Sigma) and transferred in a single 100 μl volume of fresh medium onto an 18 mm glass coverslip affixed to the center of a new 35 mm Petri dish with a small drop of Matrigel (BD Biosciences). The Petri dish contained an additional 900 μl of medium and the coverslip had been acid-washed, pre-coated with 0.1% (w/v) poly-L-lysine (Sigma) [21], and subsequently coated with substrate proteins. After the neural tubes were positioned on the substrate (ideally lying flat on one side), 500 μl of medium was immediately withdrawn from the dish to strand them in place. The dish was then carefully transferred to a tissue culture incubator (5% CO_2_, 37°C) and left undisturbed for 1 hour to give the neural tubes a chance to adhere to the substrate, after which the dish was topped up with 500 μl of fresh medium and the neural tubes were left to incubate overnight.

### 4.4 Staining of cultured cranial neural crest cells with Fc fusion proteins

The protocol used for staining cultured CNC cells with Fc fusion proteins was adapted from Prin et al. [22]. Stage 10–12 neural tube explants were grown overnight in culture on a FN-coated coverslip. The next day, the neural tubes and the cells that had grown out from them were blocked with culture medium containing 1 mg/ml bovine serum albumin (BSA) (Sigma) for 30 minutes in a tissue culture incubator (5% CO_2_, 37°C). Following a brief washing step with Ringer's solution, the cells were incubated for 1 hour at 4°C in fresh Ringer's containing 1 mg/ml BSA and 10 μg/ml of the Fc domain of human IgG or a chimeric protein made up of the extracellular domain of mouse ephrin-B1 or EphB2 fused to the Fc domain of human IgG (R&D Systems). The cells were washed, fixed with 2% paraformaldehyde in Ringer's for 10 minutes, and washed again. Finally, the cells were incubated with FITC-conjugated anti-human IgG (Sigma) diluted 1:200 in Ringer's with 1 mg/ml BSA for 30 minutes, washed, briefly incubated in DAPI nuclear stain in Ringer's (1 ng/ml), and washed again. The stained cells were then prepared for imaging by being inverted onto a drop of mounting medium on a microscope slide.

### 4.5 Preparation of stripe assay substrates

Stripe assay substrates were prepared with purified proteins as in Hornberger et al. [23] and Weinl et al. [24]. The template matrix used for applying stripes of protein to culture substrates was supplied by S. Lang [25]. Ephrin-B1 or EphB2 Fc fusion protein or Fc protein alone at the concentrations indicated in the results section was pre-clustered for 1 hour with 10X anti-human IgG (Sigma) and 110 μg/ml of an FITC marker conjugated to BSA (Sigma). A Poly-L-lysine-coated coverslip was then firmly pressed onto the stripe assay template and the Fc protein solution was drawn into the channel system with a micropipette. After 1 hour in a tissue culture incubator (5% CO_2_, 37°C), the channels were flushed with PBS and the coverslip was gently removed from the template and affixed stripe side up to the center of a 35 mm Petri dish with a drop of Matrigel. The coverslip was then blanketed with a solution of 50 mg/ml FN (Sigma) in PBS and left again for 1 hour in a tissue culture incubator. Once neural tube explants were ready to be transferred to the dish, the coverslip was rinsed with PBS and the dish was flooded with 900 μl of culture medium. The neural tube explants were positioned on these substrates such that they were perpendicular with the stripes.

For the soluble competitor experiments, soluble Fc proteins were added to a final concentration of 10 μg/ml after the stranding step. With the ephrin-B1/Fc substrate, either soluble ephrin-B1/Fc or soluble Fc alone was added. Likewise with the EphB2/Fc substrate, either soluble EphB2/Fc or soluble Fc alone was added. The medium added to the dish after the neural tube explants had been given time to attach to the substrate also contained 10 μg/ml of soluble Fc protein.

### 4.6 Antibody labeling and quantification of outgrowth cultures on stripe assay substrates

Neural tube explant cultures that showed exuberant cell outgrowth on stripe assay substrates after the overnight incubation period were processed for immunolocalization of HNK-1. Cultures were rinsed with PBS and fixed in ice-cold methanol for 10 minutes. Fixed cultures were rinsed successively with PBS and TBST, then incubated in blocking buffer (5% FBS in TBST). After 1 hour, the blocking buffer was replaced with HNK-1 antibody diluted 1:100 in fresh blocking buffer and the cultures were incubated in a humidified chamber at 4°C overnight. In some cases where the signal from the FITC-BSA marking the Fc protein stripes was weak, FITC-conjugated anti-human IgG was also added (1:100 dilution) to make the stripes more apparent. The next day, the cultures were rinsed thoroughly with TBST and incubated with secondary antibody diluted to 1:400 in blocking buffer. After 90 minutes, cultures were again rinsed thoroughly with TBST and incubated briefly with DAPI in TBST (1 ng/ml). The coverslip on which the explants were cultured was then gently removed from the dish and inverted onto a drop of mounting medium on a microscope slide.

For any given field of cell outgrowth, images were taken for the HNK-1 and DAPI signals as well as the underlying FITC-marked protein stripes. Layered composites of these images were assembled in Adobe Photoshop 6.0.1 (Adobe Systems Inc.) such that the total HNK-1-positive cell outgrowth was represented in each composite. The HNK-1 layer was used to identify and remove HNK-1-negative cells (i.e. those cells labeled by DAPI alone) as well as HNK-1-positive cells that were extensively intermingled with HNK-1-negative ones. The remaining cells in the DAPI layer were then divided according to their position relative to the FITC-marked stripes, resulting in a set of two images for each explant representing outgrowth on the stripes and outgrowth between the stripes. Cell counts were obtained with Image-Pro Plus (Media Cybernetics, Inc.), converted to percentages of the total outgrowth, averaged for each treatment, and graphed with GraphPad Prism version 4 (GraphPad Software, Inc.). Statistical analyses (standard error calculations and Fisher's exact tests) were done with GraphPad InStat (GraphPad Software, Inc.).

### 4.7 Image capturing and figure preparation

in situ hybridization images were captured with a Zeiss epifluorescence microscope (Carl Zeiss, Inc.) and DAGE-MTI 3CCD camera (DAGE-MTI, Inc.) using Scion Image 4.0.3 (Scion Corporation). Cell culture images were captured with a Leica DM 6000 B epifluorescence microscope (Leica Microsystems) and Hamamatsu Orca-ER camera (Hamamatsu Photonics) using Openlab 4.0.4 (Improvision Ltd.). Images were captured as tif files and imported into Adobe Photoshop, where they were adjusted (e.g. brightness/contrast enhancement, cropping) and assembled into figures.

## Authors' contributions

DOM did all of the experiments and prepared the first draft of the paper. RDB conceived of the study, assisted in interpreting data, and revised the manuscript. All authors read and approved the final manuscript.
